# Self-Healable and Recyclable Dual-Shape Memory Liquid Metal–Elastomer Composites

**DOI:** 10.3390/polym14112259

**Published:** 2022-06-01

**Authors:** Xiaobo Deng, Guokang Chen, Yifan Liao, Xi Lu, Shuangyan Hu, Tiansheng Gan, Stephan Handschuh-Wang, Xueli Zhang

**Affiliations:** 1 College of Chemistry and Environmental Engineering, Shenzhen University, Shenzhen 518060, China; 1900221020@email.szu.edu.cn (X.D.); cgk@szu.edu.cn (G.C.); xlu@szu.edu.cn (X.L.); shuangyan_hu@szu.edu.cn (S.H.); gantiansheng@szu.edu.cn (T.G.); stephan@szu.edu.cn (S.H.-W.); 2 Shenzhen Middle School, Shenzhen 518001, China; millerliao2019@outlook.com

**Keywords:** liquid metals, polyurethane elastomers, shape memory, recyclable electronics, self-healing

## Abstract

Liquid metal (LM)–polymer composites that combine the thermal and electrical conductivity of LMs with the shape-morphing capability of polymers are attracting a great deal of attention in the fields of reconfigurable electronics and soft robotics. However, investigation of the synergetic effect between the shape-changing properties of LMs and polymer matrices is lacking. Herein, a self-healable and recyclable dual-shape memory composite, comprising an LM (gallium) and a Diels–Alder (DA) crosslinked crystalline polyurethane (PU) elastomer, is reported. The composite exhibits a bilayer structure and achieves excellent shape programming abilities, due to the phase transitions of the LM and the crystalline PU elastomers. To demonstrate these shape-morphing abilities, a heat-triggered soft gripper, which can grasp and release objects according to the environmental temperature, is designed and built. Similarly, combining the electrical conductivity and the dual-shape memory effect of the composite, a light-controlled reconfigurable switch for a circuit is produced. In addition, due to the reversible nature of DA bonds, the composite is self-healable and recyclable. Both the LM and PU elastomer are recyclable, demonstrating the extremely high recycling efficiency (up to 96.7%) of the LM, as well as similar mechanical properties between the reprocessed elastomers and the pristine ones.

## 1. Introduction

Nature has served as the inspiration for copious inventions, by virtue of well-coordinated engineering design, material design, and continual evolution. This holds especially true for the fields of robotics and soft robots, and humankind has mimicked ample design concepts to achieve abilities and adaptabilities that resemble those of natural living systems [1]. These robots can run with high flexibility and adapt to complex environments, such as narrow gaps, or even inside the human body, which holds great promise in fields ranging from manufacturing to medicine, i.e., drug delivery, treatment, and diagnostics [2,3,4]. Generally, soft robots are built with compliant materials, including fluids, gels, and elastomers, which exhibit similar elastic moduli to the materials found in living creatures [5,6]. Recently, gallium-based room temperature liquid metals (LMs) have emerged as attractive materials for the design and production of soft robotic systems, owing to their excellent flexibility, intriguing shape-shifting abilities, and negligible toxicity [7,8,9]. The pristine liquid metal (without an oxide skin) exhibits a high surface tension (SFT, ~600 mN/m for galinstan) and, in contact with water, a high interface tension (IFT) of ~490 mN/m [10,11]. Surface oxidation is known to reduce the SFT (and IFT) of the LM considerably: the SFT of oxidized galinstan is about 360 mN/m and the IFT in water is even lower [12]. Notably, the IFT can be lowered to near 0 mN/m by applying voltage to oxidize the LM in an electrolyte solution [13]. This electrochemically tunable IFT can trigger the expansion and contraction of LM droplets in sodium hydroxide (NaOH) solution, a technique that has been employed to mimic the heartbeat, as well as for muscle contractions [14,15]. In addition, the tunable IFT can serve as the driving force for LM droplet-based soft robots, fabricated by encapsulating the LM droplets and electrolyte solutions in a closed system [16,17]. However, the forces exerted by IFT-induced movement are rather small. Therefore, other strategies for liquid metal soft robot actuation have been developed, which are based on internal fuels, ultrasound, and magnetic field actuation [18,19,20,21,22]. Although many LM droplet-enabled soft robotic systems have been reported and applied in copious areas, such as microfluidic pumping, cargo transportation, and drug delivery, they are still limited by the fact that they have fewer shape-shifting patterns and small actuation strains.

In addition to LM droplet-based soft robots, combining LM with soft polymers is of great interest to soft robotics [23,24,25]. Generally, soft polymers embedded with rigid particle fillers exhibit high stiffness and low stretchability [26,27]. In contrast, LM is an attractive liquid inclusion material for polymer modification, and facilitates the production of functional soft composites that possess intriguing mechanical, thermal, and electrical properties [28]. In the LM–polymer composites, soft polymers offer large actuation strains and various shape changes, while LMs can improve the thermal and electrical conductivities of the polymer matrix without significantly altering its mechanical properties, which is favorable for the fabrication of electrothermally powered soft actuation systems [29]. For example, incorporating LMs into liquid crystal elastomers (LCEs) can enable multifunctional soft actuators [30,31,32,33,34]. In these actuators, the LMs can form conductive paths and generate joule heat in order to induce the phase transition of LCEs, leading to shape-shifting in the LM-LCE composites. Importantly, due to their intrinsic fluidity (in their liquid state) [35,36], LMs can deform synchronously with the composite, rather than constrain the shape morphing of the LCE as is the case with solid conductive fillers, endowing the actuators with high flexibility and large actuation strains [29]. In addition, the enhanced thermal conductivity [37,38] and negligible change in the electrical conductivity during deformation [39,40] facilitate the efficient and smooth functioning of the electrothermally powered actuators. Based on these advantages, various LM–LCE soft actuation systems have been produced by applying different strategies, such as magnetic printing of LM on LCE substrates [30], 4D printing of LM–LCE composites [31], and extrusion-based direct ink writing for LM–LCE fibers [32].

Furthermore, owing to the low melting point of LMs, the solid-to-liquid phase transition can be employed to tune the electrical and mechanical properties of the LM–polymer composites, and to realize the shape programming of LM–polymer composites at or near room temperature. For the polymer composites embedded with LM droplets, the LM droplets expand upon solidification (freezing) [41]. This property can be used to render an insulating composite reversibly conductive. In addition, shape reconfiguration of the composites can be achieved through the melting and solidification of LM droplets. The compliant LM droplets allow the composites to be easily programed into a new shape, while the solidification of the LM droplets reinforces the composites and fixes the new shape [42]. By finely tuning the distribution of LM droplets in the polymer matrix, the shape-morphing properties of the composites can be improved. For example, bilayer-structured LM droplet–elastomer composites have been fabricated by the gravitational sedimentation of LM droplets during the curing of the elastomers [43]. The phase transition of the LM droplets caused a mechanical mismatch between the LM-rich layer and the elastomer-rich layer, endowing the resulting composites with a thermal- or light-induced shape memory effect. Similarly, LM–elastomer composite fibers with gradient-dispersed LM droplets have been reported; these exhibited reversible shape programmability, enabled by the phase transition of the LM droplets [44]. However, in these cases, the shape morphing of the composites is only governed by the LM droplets, and the polymer matrix did not show a shape changing ability, which limits the composites’ potential for complex shape programming, and therefore limits their broader applications.

Here, we report the fabrication of multifunctional LM–polymer composites with dual-shape memory abilities and self-healing properties. Moreover, the composite can be reused (reprocessed) and, after its obsolescence, the polymer and the liquid metal can be recycled with high efficiency. The composites comprise gallium (Ga, melting temperature 29.4 °C) and a polyurethane (PU) elastomer, and are fabricated via a sedimentation technique, leading to the formation of a bilayer structure. Notably, the PU elastomers contain crystalline regions characterized by a melting point of around 40.0 °C. The phase transitions of the gallium (microdroplets) and PU elastomer regulate the mechanical properties of the gallium-rich layer and the elastomer layer, respectively, enabling complex shape programming of the Ga–PU composites. The shape morphing can be programmed into the composites by virtue of the two different crystalline temperatures. The shape recovery is dominated by the two different melting temperatures. To demonstrate this, we employed the shape-morphing composites to design and fabricate a soft gripper that can grasp and release objects. Similarly, we employed the composite as a light-controlled reconfigurable switch for a circuit. Moreover, the PU elastomers are crosslinked by Diels–Alder (DA) bonds, which dissociate at a high temperature (~120 °C) and reform at a low temperature (<70 °C) [45], endowing the composite with self-healing abilities and recyclability. Both gallium and PU elastomers are recyclable, and the recycling efficiency of gallium is 96.7%. Moreover, the PU elastomers can be reprocessed, and the reprocessed polymer shows comparable mechanical properties to the original one.

## 2. Materials and Methods

### 2.1. Materials

Polycaprolactone diol (PCL-diol, M_n_ = 2000 g/mol) was purchased from Sigma-Aldrich (St. Louis, MO, USA). Gallium (Ga, melting point: 29.4 °C), 1,6-hexamethylene diisocyanate (HDI, 99%), 4,4’-bismaleimidodiphenylmethane (BMI, 96%), 2,5-furandimethanol (Fu-diol, 98%), anhydrous N, N-dimethylformamide (DMF, 99.8%), and dibutyltindilaurate (DBTDL, 95%) were purchased from Aladdin (Shanghai, China). All reagents were used as received, without further purification, unless otherwise noted.

### 2.2. Synthesis of PU Prepolymers

PCL-diol and Fu-diol were vacuum dried (80 °C) overnight before use. PCL-diol (2 g, 1 mmol), Fu-diol (0.128 g, 1 mmol), and anhydrous DMF (15 mL) were mixed in a glass vial by stirring for 5 min. Then, HDI (0.336 g, 2 mmol) and DBTDL (3 droplets) were added into the glass vial. The reaction commenced at room temperature and continued for 24 h. Afterwards, a solution comprising 0.179 g (0.5 mmol) BMI and 1,4-hydroxy benzene (3.0 wt% of BMI) in 5 mL DMF was prepared, and added to the vial [46]. Subsequently, the resulting mixture was concentrated to a viscous solution by using a rotary evaporator, operating at 65 °C for 10 min (removing most of the solvent), and the PU prepolymers were obtained.

### 2.3. Preparation of Bilayer-Structured Ga–PU Composites

First, Ga was liquefied at 60 °C for 30 min, and mixed with the PU prepolymers by mechanical agitation. During the mixing process, liquid Ga was broken into microdroplets, which were dispersed into the prepolymers. The resulting mixture was poured into a Teflon mold or another non-stick mold, and degassed in vacuo. Afterwards, the mixture was solidified at 60 °C in an oven. In the meantime, the Ga microdroplets settled down. After 48 h, the Ga–PU composite with a bilayer structure was obtained.

### 2.4. Characterization

The morphology of the Ga–PU composites was recorded on a scanning electron microscopy (SEM) (APREO S, ThermoFisher Scientific, Waltham, MA, USA) coupled with an energy dispersive X-ray spectroscopy (EDS). The SEM accelerating voltage was 5 kV. To acquire the cross-sectional SEM images, the samples were prepared by fracturing in liquid nitrogen. The thickness of the gallium-rich layer was measured at three different positions by analyzing the cross-sectional SEM image of one sample, and the results were averaged. The Fourier transform infrared (FT-IR) spectra were recorded on an IR Affinity-1 (Shimadzu, Kyoto, Japan) spectrometer. The phase transitions of the Ga–PU composites were measured using a differential scanning calorimeter (DSC, DSC-200F3, NETZSCH, Selb, Germany) in the temperature range of −80 °C to 100 °C, at a rate of 10 °C min^−1^ under a nitrogen atmosphere (flow rate: 50 mL min^−1^), and the data were recorded after the first heating scan that eliminated the thermal history. The sheet resistance was measured by a four-probe method using a Keithley 2400 sourcemeter (Tektronix, Beaverton, OR, USA). Three samples were examined for each Ga–PU composite, and the average was taken. The temperature and IR image of the Ga–PU composites were recorded by an IR camera (FLIR ONE Pro, Beijing Justsun Science & Technology, Beijing, China). Tensile tests were performed using a CMT6103 electronic universal testing machine (SANS, Shenzhen, China). The tests were conducted at room temperature at a speed of 10 mm/min. Three samples were examined to calculate the elastic modulus and the strain at break for each Ga–PU composite. The molecular weight of the PU prepolymer was measured by a gel permeation chromatography (GPC) system (Waters 515, Waters Corporation, Milford, MA, USA), with polystyrene as a standard and DMF as eluent (solvent), at a flow rate of 1.0 mL min^−1^.

The tests of the gel fraction and swelling ratio of the composites were executed as follows: a small piece of the Ga–PU composite sample was immersed into DMF at room temperature for 48 h. The masses of the original sample (*m*_0_), the swelled sample (*m*_1_), and the dried sample (*m*_2_) were measured. The gel fraction (G (%)) and the swelling ratio (S (%)) were calculated according to Equations 1 and 2, respectively. Three samples were examined for calculating the G (%) and S (%) for each Ga–PU composite.

(1) $$G\left(\%\right)=\frac{m_{2}}{m_{0}}\times 100\%$$

(2) $$S\left(\%\right)=\frac{m_{1}}{m_{2}}\times 100\%$$

To demonstrate the shape memory behavior of the Ga–PU composites, a strip of Ga–PU composite (50 × 8 × 1 mm^3^) was heated to 65 °C to melt both the Ga and the PU elastomer, followed by bending at a given angle (90°) (Figure S1). Afterwards, the strip was quenched at −10 °C to fix it in the first temporary shape (S1) by crystallization of PU elastomer, and the angle *α* was recorded. Subsequently, the strip sample was continuously bent to another given angle (180°) and quenched at −35 °C to fix the second temporary shape (S2). The angle *β* was recorded. Thereafter, the programmed strip-like sample was first heated to 35 °C to recover its shape to the first temporary shape (S1) and the angle *θ* was also recorded. By heating to 65 °C, the composite recovered its original shape, and the angle *γ* was recorded. The shape fixity ratio and shape recovery ratio were calculated according to Equations 3 to 6. Three samples were examined for calculating the shape fixity ratio and shape recovery ratio for each Ga–PU composite.

(3) $$F_{S0\to S1}=\frac{180{}^{\circ}-\alpha }{90{}^{\circ}}\times 100\%$$

(4) $$F_{S1\to S2}=\frac{\alpha -\beta }{\alpha }\times 100\%$$

(5) $$R_{S2\to S1}=\frac{\theta -\beta }{\alpha -\beta }\times 100\%$$

(6) $$R_{S1\to S0}=\frac{\gamma -\theta }{180{}^{\circ}-\theta }\times 100\%$$

## 3. Results and Discussion

### 3.1. Fabrication and Characterization of the Ga–PU Composites

**Figure 1:**
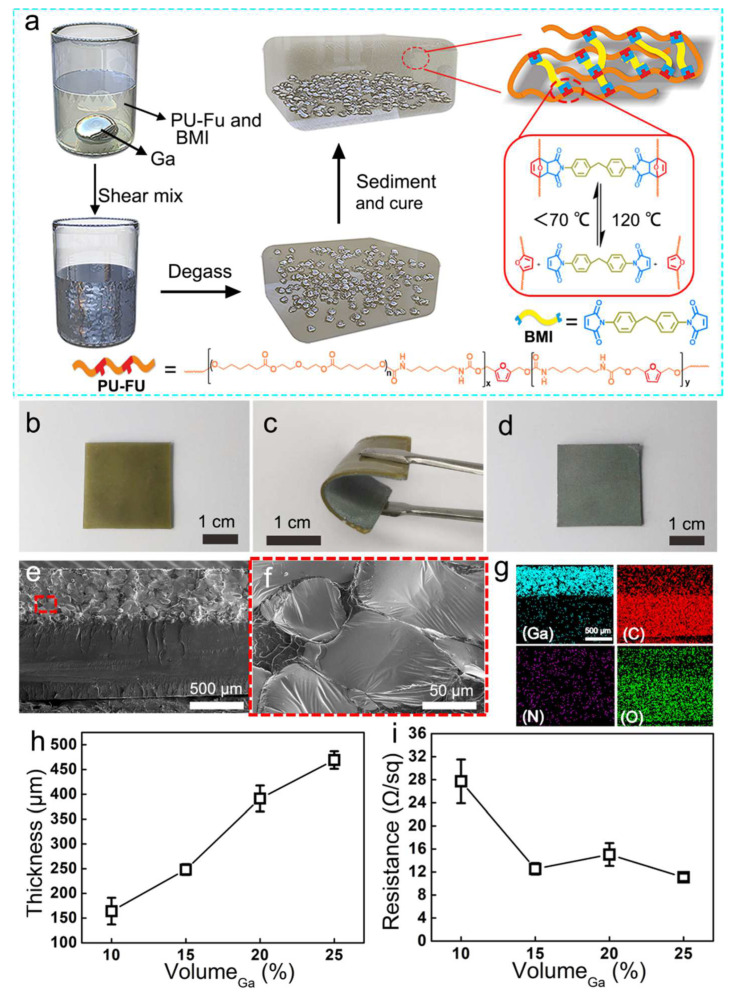
Fabrication and characterization of bilayer-structured Ga–PU composites. (**a**) Schematic illustration of the fabrication procedure of the composites, together with a schematic depiction of the elastomer structure and the mechanism of the formation and dissolution of the dynamic DA bonds. (**b**–**d**) Optical photographs of the as-fabricated Ga–PU composite (15 vol% Ga): (**b**) elastomer-rich layer, (**c**) flexibility of the composite, and (**d**) Ga-rich layer. (**e**–**f**) SEM micrographs and (**g**) EDS mapping of the cross-section of one representative composite (25 vol% Ga); the upper area is the Ga-rich phase of the composite. (**h**) Thickness of the Ga-rich layer and (**i**) sheet resistance of the Ga–PU composites dependent on the vol% of Ga.

The preparation of the bilayer Ga–PU composites is illustrated in Figure 1a. PU prepolymers containing furan (Fu) group were synthesized from polycaprolactone diol (PCL-diol), hexamethylene diisocyanate (HDI), and 2,5-furandimethanol (Fu-diol) through polycondensation (Scheme S1). The resulting PU prepolymer exhibited a number average molecular weight of 6.6 × 104 g/mol (Figure S2). Afterwards, liquid gallium, N-dimethylformamide (DMF) solution of PU prepolymer, and crosslinker (4,4’-bismaleimidodiphenylmethane, BMI) were blended by shear mixing, resulting in the formation of a viscous suspension of Ga microdroplets. The resulting mixture was poured into a Teflon mold, followed by degassing, and curing at 60 °C for 48 h. During the curing process, the Ga microdroplets sedimented, the DMF evaporated, and the PU prepolymer was crosslinked by BMI via the DA reaction. The as-prepared LM–PU composite is flexible and exhibits a bilayer structure (Figure 1b–d). The Fourier transform infrared (FT-IR) spectra of the composite demonstrates the near completion of the DA reaction. The absorption band for the DA bond at 871 cm^−1^ emerges in the FT-IR spectra of the composite, while the absorption bands for maleimide at 837 and 689 cm^−1^, and for the furan ring at 1008 cm^−1^, almost vanish (Figure S3) [46,47,48]. In addition, the formation of such a DA reaction-based crosslinking network was further confirmed by a swelling test (Figure S4).

The cross-section of the Ga–PU composite was analyzed by scanning electron microscopy (SEM) and energy-dispersive X-ray spectroscopy (EDS). The SEM image shows that the composite is divided into two layers, namely, the pure PU elastomer and the enriched Ga microdroplet layer (Figure 1e). The Ga microdroplets are 50 to 100 µm in diameter (Figure 1f and Figure S5). The EDS mapping illustrates the hierarchical distribution of Ga and C elements, further confirming the discernable bilayer structure of the Ga–PU composites (Figure 1g). The thickness of the Ga-rich layer is strongly dependent on the Ga content. As the vol% of Ga increases from 10% to 25%, the thickness of the Ga-rich layer increases nearly linearly from 150 μm to 500 μm (Figure 1h). In the Ga-rich layer, Ga microdroplets are in close proximity to each other and form a percolation network, endowing the Ga–PU composites with electrical conductivity. In previous studies, a high LM content (>30 vol%) and a mechanical sintering process were often required to realize electrical conductivity in LM polymer composites [49,50,51,52,53,54]. By comparison, the present bilayer-structured Ga–PU composites can acquire good electrical conductivity at a relatively low Ga content without necessitating a mechanical sintering process. The sheet resistance of the Ga–PU composites is 28 Ω/sq at 10 vol% of Ga, and decreases to 12 Ω/sq at 15 vol% Ga (Figure 1i). By further increasing the Ga content to 20 and 25 vol%, the sheet resistance of the composites barely changes. After storage at ambient conditions for 30 days, the sheet resistance of the composite with 25 vol% Ga slightly increases to 18 Ω/sq, which may be attributed to the oxidation of the Ga (Figure S6).

### 3.2. Dual-Shape Memory Effect of the Ga–PU Composites

**Figure 2:**
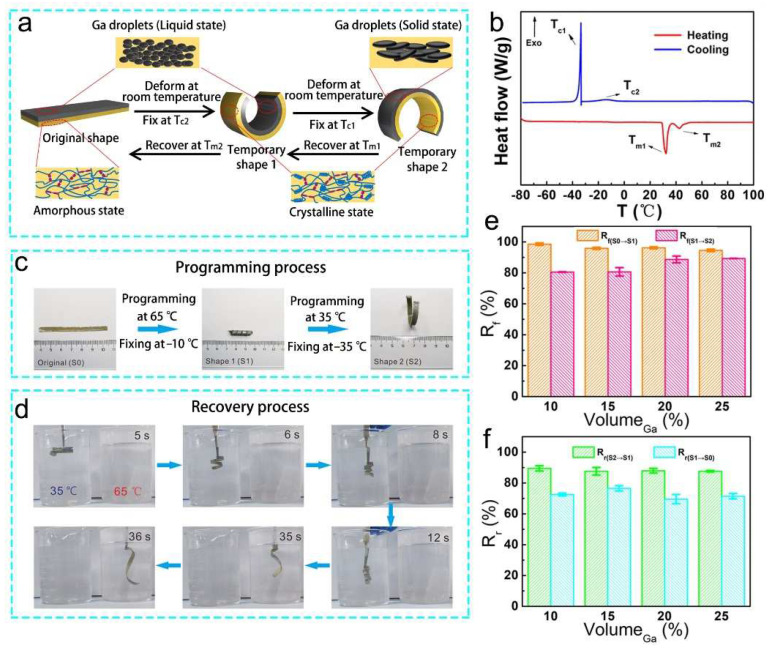
Shape morphing of the Ga–PU composites. (**a**) The schematic depiction of the mechanism of the dual-shape memory effect of the composite. (**b**) DSC curves of the composite (20 vol% Ga). (**c**–**d**) Photographs showing (**c**) the programming and (**d**) the recovery process of the dual-shape memory composite (20 vol% Ga). (**e**–**f**) The fixing ratio and recovery ratio for the shape change of the composites.

The thermal behavior of the Ga–PU composite was characterized by differential scanning calorimetry (DSC). As shown in Figure 2b, the Ga–PU composite shows two melting temperatures in the DSC heating curve, and two crystallization temperatures in the DSC cooling curve. In the composite, the Ga microdroplets crystallize at a much lower temperature (–33.3 °C) during the cooling process, due to the supercooling and size effects [55,56], while melting is observed at 29.4 °C during the heating process. The other peak, at around −10.0 °C in the cooling curve, is attributed to the crystallization of PCL segments in the PU elastomer. The resulting PCL crystalline region melts at 40.0 °C in the heating curve, which is slightly lower than the melting point of PCL-diol (43.0 °C, Figure S7). Accordingly, the mechanical properties of Ga–PU composites strongly depend on the crystallization of Ga microdroplets and PCL segments (Figure S8). The Ga–PU composites possess a lower elastic modulus and smaller strain at break than the pure PU elastomer, because the LM-rich layer is weakened by the high loading of Ga. As both Ga microdroplets and PCL segments crystalize, the composites are stiff with elastic moduli ranging between 121.3 and 130.6 MPa. The stiff composites transform into a semisoft state with lower moduli between 23.4 and 32.3 MPa as the crystalline Ga microparticles melt. By melting the crystalline region in the PU elastomer, the composites become soft and stretchable, and possess moduli between 0.5 and 1.2 MPa and an average maximum extension larger than 500%.

The bilayer structure, as well as the phase transitions of Ga and PU elastomers, endow the Ga–PU composites with excellent shape-morphing capabilities. Pure PU elastomers can be programmed into a curled shape by crystallization, and recover their original shape upon melting (Figure S9). More importantly, the synergistic action of the phase transitions of Ga and PU elastomer enables the dual-shape memory effect of the Ga–PU composites. The strategy and mechanism for shape morphing of the composites are illustrated in Figure 2a. As a demonstration, a strip of the composite is heated to 65 °C to liquefy the Ga microparticles and transition the PU elastomer from a glassy to a rubbery state. Then, it is deformed into a curled shape (temporary shape 1, S1) (Figure 2c). The curled shape is fixed by freezing at −10 °C to trigger the crystallization of PCL segments. Subsequently, the curled sample is folded into a circle-like structure (temporary shape 2, S2) and fixed at −35 °C, where the Ga microdroplets solidify. Interestingly, the shape recovery can be realized by gradually melting the solid Ga and the crystalline region in the PU elastomer. When heated to 35 °C, the solid Ga melts, and the circular-shaped sample recovers to the previous curled shape (S1) (Figure 2d and Video S1). The driving forces for regaining the previous shape are the stored energy in the elastomer and the stress release in the Ga-rich layer. By further heating to 65 °C, melting of the crystalline regions in the PU elastomer is induced, and the curled-shape sample recovers its original shape, owing to the stress release in the elastomer.

To further evaluate the shape-morphing efficiency of the Ga–PU composites, a bending test (Figure S1) was performed to calculate the shape fixity ratio (R_f_) and shape recovery ratio (R_r_) of the composites [57,58,59]. As shown in Figure 2e,f, the composites exhibit a good shape memory performance, with the shape fixity ratio beyond 80% and the shape recovery ratio beyond 70%. According to the shape-morphing mechanism, the crystallization of PCL segments in the PU elastomer plays the main role in the fixing of the first temporary shape (S1), while the solidification of LM microdroplets is primarily responsible for the fixing of the second temporary shape (S2). Therefore, by increasing the content of Ga, R_f(S0→S1)_ decreases while R_f(S1→S2)_ increases slightly. In particular, both R_f(S0→S1)_ and *R*_f(S1→S2)_ are higher than 90% for the composite with 25 vol% Ga. In addition, R_f(S0→S1)_ is always larger than R_f(S1→S2)_ for all Ga loadings. This is because the crystallization of the PCL segments enhances the mechanical properties of the composites better than the solidification of Ga microdroplets (Figure S8). Similarly, during the recovery process, R_r(S2→S1)_ is always larger than R_r(S1→S0)_ for all Ga loadings. The melting of solidified Ga governs the shape transformation from S2 to S1, while the melting of the PCL crystalline regions dominates the transformation from S1 to S0. Eventually, the composite becomes soft and deformable, and its low modulus slightly weakens its ability to recover its original shape [60].

**Figure 3:**
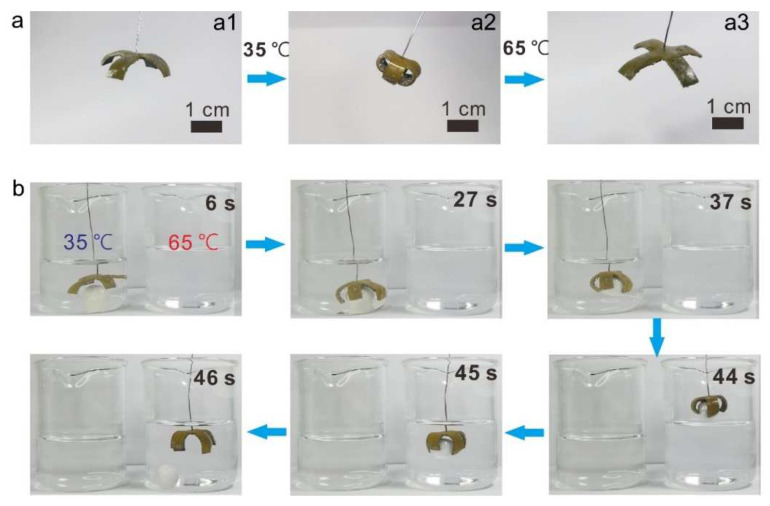
A soft gripper fabricated with the Ga–PU composite (20 vol% Ga). (**a**) The programed gripper undergoes shape change in response to temperature variation. (**b**) The gripper grasps and transfers an object.

Based on the composite’s excellent shape-morphing performance and dual-shape memory abilities, a four-fingered gripper is designed and constructed using the LM–PU composites (Figure 3a). To make this gripper work, a shape programming process is performed. Specifically, the gripper, which is initially in an open state (Figure 3(a1)), is folded into a clenched state (Figure 3(a2)) as the first temporary shape. Subsequently, the gripper shape is fixed by the crystallization of PCL segments at −10 °C. The clenched gripper is forced to open again to form the second temporary shape (Figure 3(a3)), which is fixed by the solidification of Ga microdroplets at −35 °C. As proof of concept, the pre-programmed gripper is employed to move an object from one beaker to another (Figure 3b and Video S2). When the gripper reaches into the first beaker filled with 35 °C water, which melts the solidified Ga microparticles, the gripper gradually recovers its clenched state and tightly grasps the ball. Subsequently, the gripper takes the ball out of the first beaker and moves it to the second beaker, which is filled with 65 °C water. The PCL crystalline regions melt in such an environment, and the gripper returns to the original shape, releasing the ball in the process.

### 3.3. Light-Controlled and Self-Healing Circuits Enabled by the Ga–PU Composites

**Figure 4:**
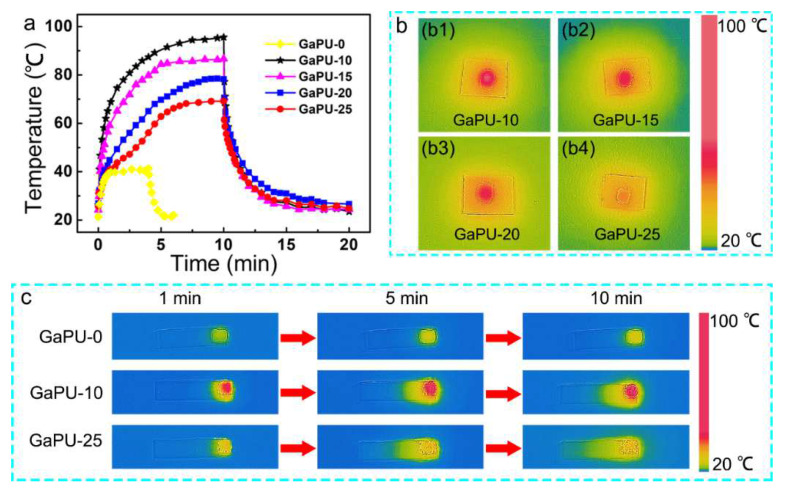
Photothermal effect of the Ga–PU composites. (**a**) The temperature as the function of time for the composite under NIR irradiation (2 W/cm^2^). (**b**) Infrared thermal images for the composites with different vol% of Ga under NIR irradiation. (**c**) Heat transport under localized NIR irradiation.

Due to the photothermal effect of Ga, remote localized heating of the Ga–PU composites can be realized by near infrared (NIR) light irradiation [43,59]. For all of the samples, the temperature at the irradiated region increases with irradiation time (Figure 4a). The pure PU elastomer shows an equilibrium temperature of 40 °C under irradiation (2 W/cm^2^). By loading 10 vol% Ga, the equilibrium temperature of the composite reaches nearly 100 °C in less than 10 min of irradiation. As the Ga content increases from 10 to 25 vol%, the heating rate gradually slows down, and the equilibrium temperature decreases to 70 °C (Figure 4a,b). This phenomenon is attributed to the high thermal conductivity of Ga [37,38]. With higher Ga loadings, the Ga-rich layer of the bilayer-structured composites exhibits higher thermal conductivity, enabling it to distribute and dissipate the thermal energy more efficiently. As shown in Figure 4c, the heat is limited to the irradiated region of the pure PU elastomer, owing to its poor thermal conductivity, while the heat transport in the Ga-rich layer occurs for composites. Accordingly, the higher Ga loadings enable faster heat transport along the LM-rich layer, and thus lower the temperature of the irradiated region.

**Figure 5:**
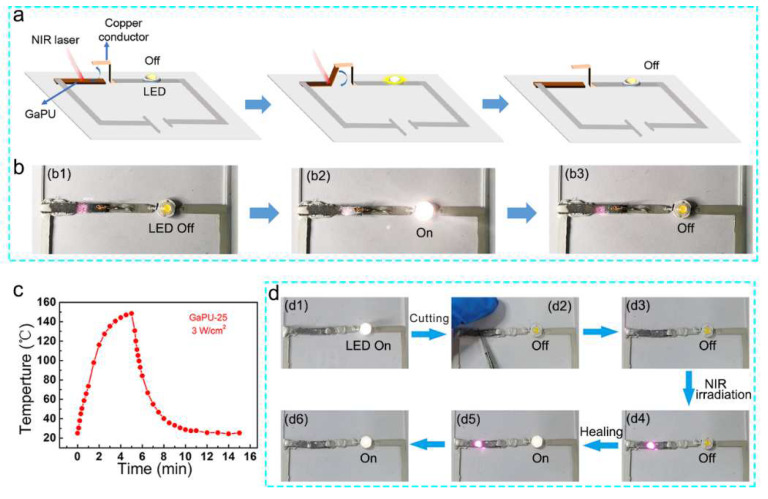
Light-controlled and self-healing LED circuits enabled by Ga–PU composites. (**a**) Schematic illustrations showing a light-controlled switch made of the composite for an electric circuit. (**b**) An LED circuit is remotely controlled by the composite switch (20 vol% Ga) under NIR light irradiation. (**c**) The temperature as a function of time for the composite with 25 vol% Ga under NIR irradiation (3 W/cm^2^). (**d**) Photographs demonstrating the self-healing LED circuit with the composite (25 vol% Ga) as conductor; the composite is severed by a knife and healed by irradiating the break with NIR light.

As the temperature of Ga–PU composites under NIR light irradiation are higher than the melting point of gallium and the PCL crystalline regions, the shape memory behavior of the composites can be controlled by light. In addition, combining the light-induced shape-memory effect and good electrical conductivity, a light-controlled light-emitting-diode (LED) circuit is designed and fabricated using the composite (Figure 5a). To realize this circuit, a composite strip is programmed into a half-folded shape (the first temporary shape), and then the half-folded strip is straightened again as the second temporary shape. The resulting composite strip is applied as a light-controlled switch, which can be used to remotely turn the LED lamp on and off by use of light-induced shape recovery. As shown in Figure 5b and Video S3, under NIR light irradiation, the straight composite strip gradually returns to the half-folded shape and makes contact with the copper conductor, and thus the LED lamp lights up. The LED lamp can be turned off again by further irradiating the composite strip to raise its temperature and trigger the second recovery process to its original straight shape.

Moreover, the Ga–PU composites can enable self-healing circuits, as DA bond-based networks in the PU elastomer can be broken at high temperatures (>120 °C) while re-forming upon cooling. To evaluate the self-healing performance, a strip of the pure PU elastomer was cut into two pieces, and then heated to 120 °C for 10 min to fuse the two pieces together. Afterwards, the merged sample was heated to 65 °C for 72 h to allow the rebuilding of DA bonds. The healed sample shows a repair efficiency of 92% for the elastic modulus, 71% for the strength at break, and 68% for the strain at break, indicating the good self-healing performance of the PU elastomer (Figure S10). To reach the threshold temperature for DA bond disassociation, NIR light with higher power density (3 W/cm^2^) is employed to heat the composites. As shown in Figure 5c, the temperature at the irradiated region increases rapidly to 120 °C within 3 min, indicating the possibility of highly localized light-induced self-healing of the composites. To demonstrate the self-healing of the composite, a simple LED circuit with the composite as a self-healable conductor is fabricated. As shown in Figure 5d and Video S4, the LED lamp emits light at the beginning. Upon severing the composite using a knife, the LED stops emitting light. By treating the gap with an NIR light, the composite melts and electrical conductivity is reestablished. Accordingly, the LED lamp lights up again.

### 3.4. Recycling of the Ga–PU Composites

**Figure 6:**
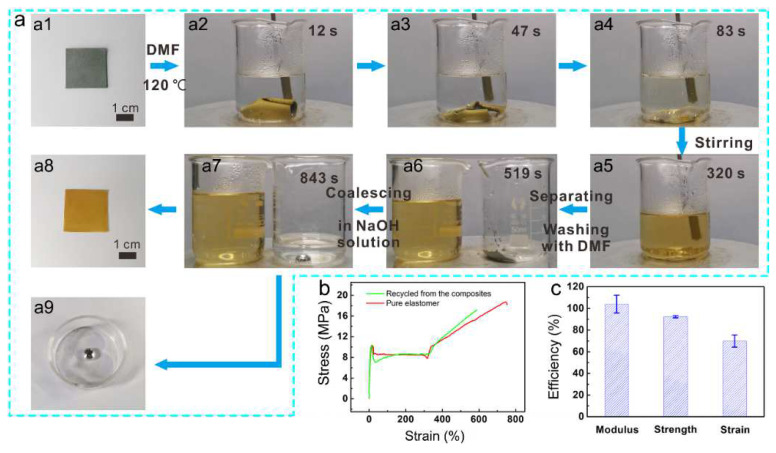
Recycling of the Ga–PU composites (25 vol% Ga). (**a**) Photographs showing the recycling processes for the LM and PU elastomer. (**b**) Typical stress–strain curves for the as-prepared (original) elastomer (red) and the reprocessed elastomer (green). (**c**) Retention (percentage) of the elastic modulus, strength at break, and strain at break upon reprocessing. The figure of 100% denotes the mechanical properties of the original elastomer.

In addition to self-healing performance, the DA bond-based network also endows the Ga–PU composites with recyclability. Notably, unlike other dynamic covalent bonds, such as dynamic ester bonds, which require a catalyst to trigger bond dissociation [61,62], DA bonds dissociate completely into furan groups and imide groups at a high temperature (ca. 120 °C). Therefore, the DA bond crosslinked polymer can be fully dissolved in solvents upon heating [46,63]. This chemical nature of DA bonds enables the separation of the Ga microdroplets and the PU elastomer. The separated Ga microdroplets are easily recycled because of their high interfacial tension in acidic or alkaline environments [64]. Accordingly, both the Ga and polymer matrix from the Ga–PU composites can be recycled, which is an important improvement compared to other electric fillers-based (e.g., carbon nanotubes [65]) composite systems, some of which are very challenging to be recycled. As shown in Figure 6a and Video S5, a Ga–PU composite film is immersed into DMF and heated to 120 °C. By stirring for 320 s, the PU elastomer component of the composite is completely dissolved, allowing the residual Ga microdroplets to precipitate at the bottom of the beaker. After washing with DMF several times, the Ga residue is treated with a base (i.e., 0.6 M NaOH aqueous solution). The oxide layer of Ga microdroplets is quickly removed, which significantly increases the interfacial tension of the Ga/base solution and provides the driving force for the Ga droplets to merge into a large LM droplet (reducing the surface area of Ga) [10,66,67]. Finally, a macroscopic Ga droplet is obtained, which can be reused readily. In this experiment, the recycling efficiency is as high as 96.7%, which is comparable to the result (~96%) obtained in a previous report where liquid metal is filled into polymer microchannels [64]. Moreover, the PU prepolymer can also be obtained by removing the DMF, and reprocessing by heating in the oven at 70 °C for 48 h, during which the prepolymer is crosslinked again via the DA reaction between the Fu groups and BMI. The recycled PU elastomer exhibits similar mechanical properties to those of the pristine elastomer (Figure 6b,c).

## 4. Conclusions

In conclusion, we have fabricated a bilayer-structured composite comprising gallium microdroplets and a DA bond crosslinked crystalline PU elastomer, via a sedimentation method. Importantly, gallium and the PU elastomer show different melting and crystalline temperatures, which can regulate the mechanical properties of the Ga–PU composites. The combination of the bilayer structure and the two different phase transitions enables complex and reliable shape programming of the composites:

- The composites exhibit high shape-morphing efficiency, with the shape fixity ratio exceeding 80% and the shape recovery ratio exceeding 70%.
- A four-fingered gripper was designed and fabricated by the shape programming of the composite; the gripper demonstrated the ability to catch and release an object.
- Combining its photothermal effect, electrical conductivity, and shape-morphing properties, the composite was used to design a light-controlled LED circuit that can remotely turn an LED lamp on and off, signifying that remote, on demand, and localized shape morphing is possible.
- The nature of DA bond crosslinked networks makes the composites self-healable and recyclable. Both Ga and PU elastomer are readily recycled, and the recycling efficiency of Ga is as high as 96.7%. The mechanical performance of the recycled PU elastomer is close to that of the pristine one.
- This multifunctional Ga–PU composite incorporates the properties of liquid metals and PU elastomers in terms of material and structural design, and exhibits enhanced performances for potential applications in soft robotics, reconfigurable electronics, and transient devices.

## Data Availability

The data presented in this study are available on reasonable request from the corresponding author.

## Supplementary Materials

The following supporting information can be downloaded at: https://www.mdpi.com/article/10.3390/polym14112259/s1, Scheme S1: schematic of the chemical synthesis of the PU prepolymer; Figure S1: schematic illustration of the definition of the released angle and the recovered angle; Figure S2: molecular weight distribution of the PU prepolymer measured by a gel permeation chromatography (GPC) system with polystyrene as a standard and DMF as eluent (solvent) at a flow rate of 1.0 mL min^−1^; Figure S3: FT-IR spectra of Fu, BMI, and PU elastomer; Figure S4: gel fraction and swelling fraction of the Ga–PU composites; Figure S5: SEM micrographs of the Ga–PU composites; Figure S6: sheet resistance of the Ga–PU composite changing with storing time; Figure S7: DSC curve of PCL-diol; Figure S8: mechanical properties of the PU elastomer and Ga–PU composites; Figure S9: shape programming and shape recovery of the pure PU elastomer; Figure S10: mechanical properties of the pristine and healed PU elastomers; Video S1: shape recovery of the programmed Ga–PU composite; Video S2: a soft gripper grasping and transferring an object; Video S3: a light-controlled switch made of Ga–PU composite for a LED circuit; Video S4: a self-healing LED circuit with Ga–PU composite as conductor; Video S5: the recycling of the LM and PU elastomer.
