# Supplementary material for: Self-Healable and Recyclable Dual-Shape Memory Liquid Metal–Elastomer Composites
Source: Polymers (Basel). 2022 Jun 1;14(11):2259. doi: 10.3390/polym14112259 (PMC9182922; doi:10.3390/polym14112259)
Supplement: Supplementary file 1 [file polymers-14-02259-s001.zip › polymers-1735787-supplementary.pdf]

Self-Healable and Recyclable Dual-Shape Memory Liquid Metal–Elastomer Composites

Xiaobo Deng [1,†], Guokang Chen [1,†], Yifan Liao [2], Xi Lu [1], Shuangyan Hu [1], Tiansheng Gan [1], Stephan Handschuh-Wang [1] and Xueli Zhang [1,*]

[1] College of Chemistry and Environmental Engineering, Shenzhen University, Shenzhen 518060, China; 1900221020@email.szu.edu.cn (X.D.); cgk@szu.edu.cn (G.C.); xlu@szu.edu.cn (X.L.); shuangyan_hu@szu.edu.cn (S.H.); gantiansheng@szu.edu.cn (T.G.); stephan@szu.edu.cn (S.H.-W.)
[2] Shenzhen Middle School, Shenzhen 518001, China; millerliao2019@outlook.com
* Correspondence: xlzhang@szu.edu.cn.
† These authors contributed equally.

Video S1: Shape recovery of the programmed Ga-PU composite.

Video S2: A soft gripper grasping and transferring an object.

Video S3: A light-controlled switch made of Ga-PU composite for a LED circuit.

Video S4: A self-healing LED circuit with Ga-PU composite as conductor.

Video S5: The recycling of the LM and PU elastomer.

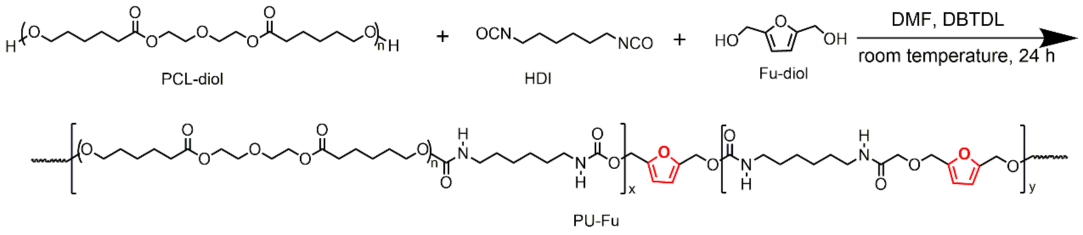

Scheme S1. Schematic of the chemical synthesis of the PU prepolymer.

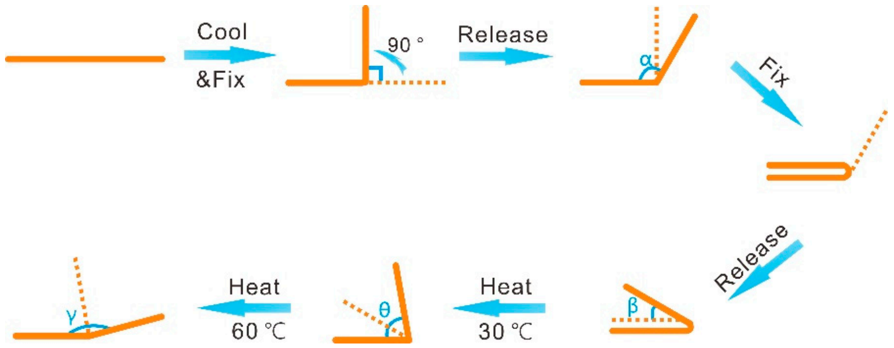

Figure S1. Schematic illustration of the definition of the released angle and the recovered angle.

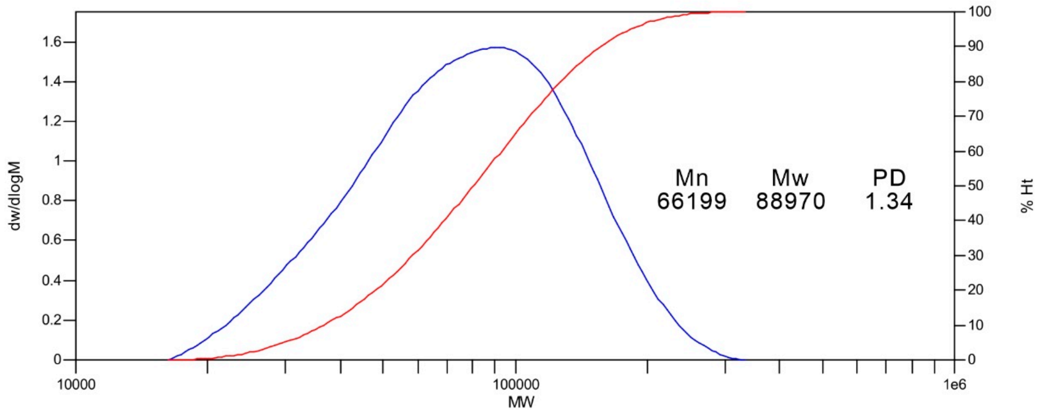

Figure S2. Molecular weight distribution of the PU prepolymer measured by a gel permeation chromatography (GPC) system with polystyrene as a standard and DMF as eluent (solvent) at a flow rate of 1.0 mL min^{-1}.

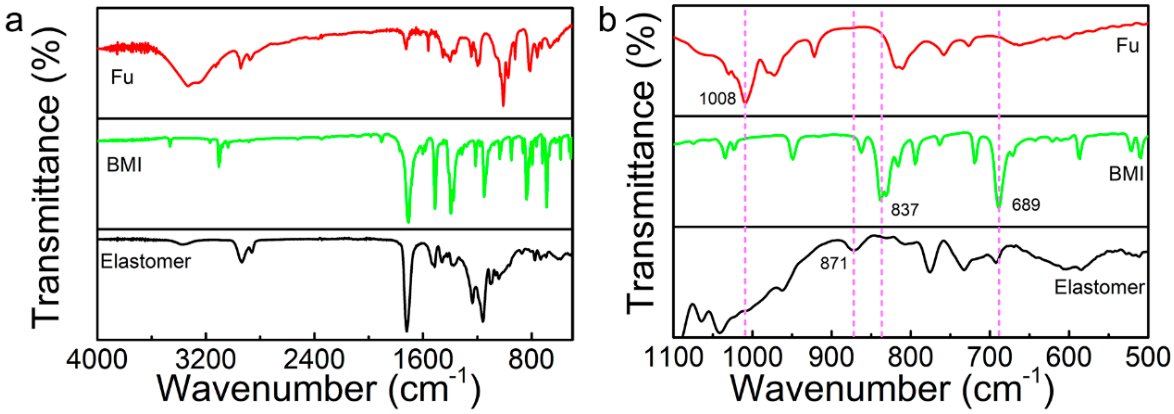

Figure S3. (a-b) The FT-IR spectra of 2,5-furandimethanol (Fu), 4,4'-bismaleimidodiphenylmethane (BMI), and PU elastomer in the range from (a) 400 to 4000 cm^{-1} and (b) 500 to 1100 cm^{-1}.

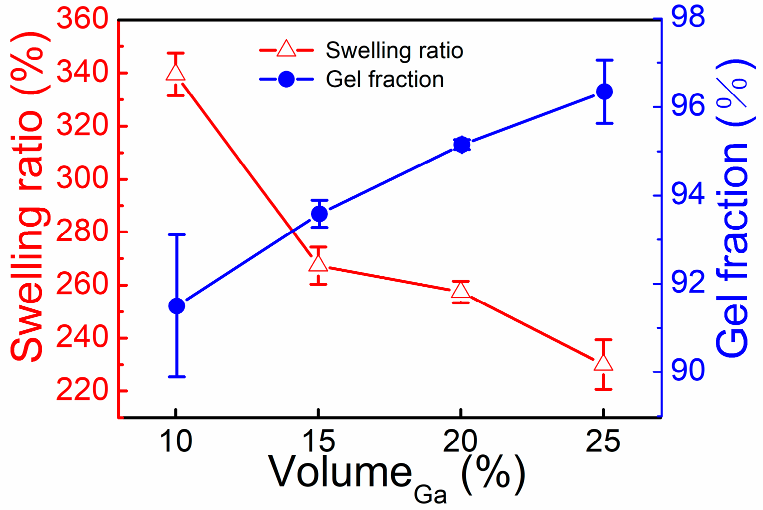

Figure S4. Gel fraction and swelling fraction of the Ga-PU composites.

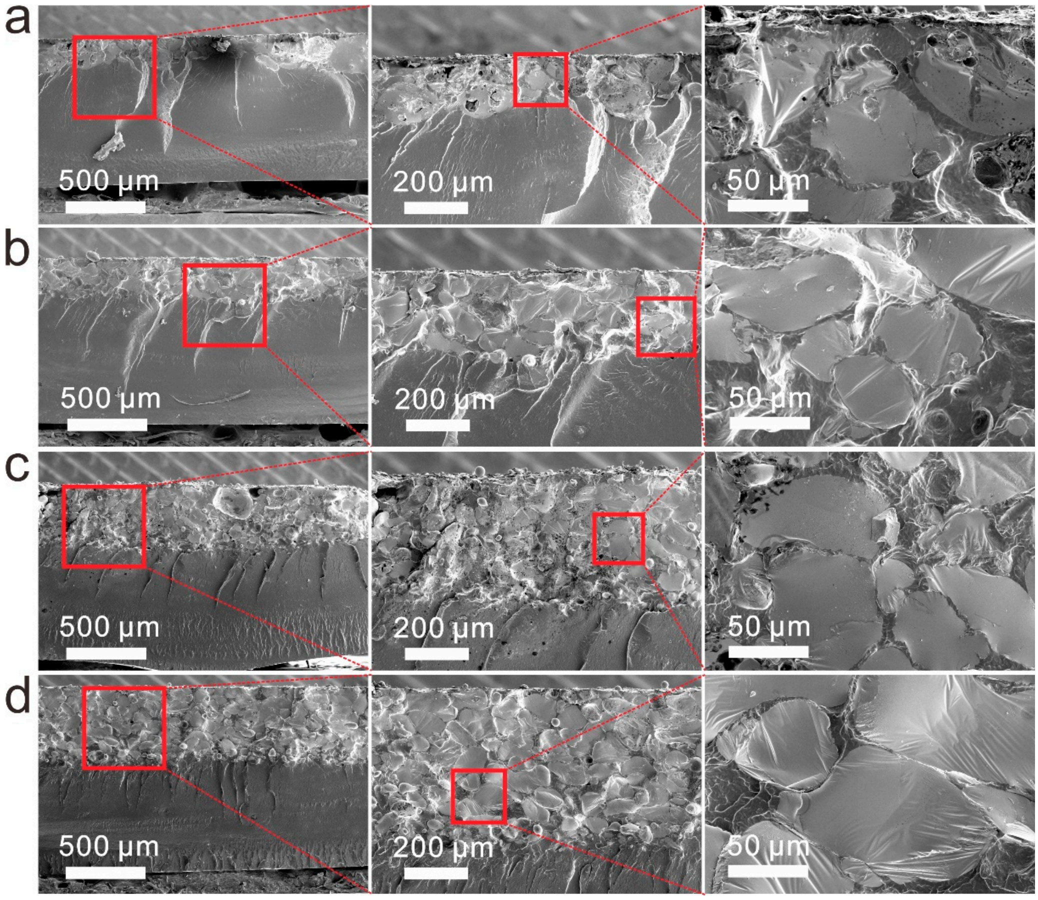

Figure S5. SEM micrographs of Ga-PU composite. The Ga-PU composites fabricated with a Ga volume ratio of (a) 10%, (b) 15%, (c) 20%, (d) 25%. The first column shows the whole composite, the second column shows the Ga-rich layer, and the third column shows a magnification of Ga droplets.

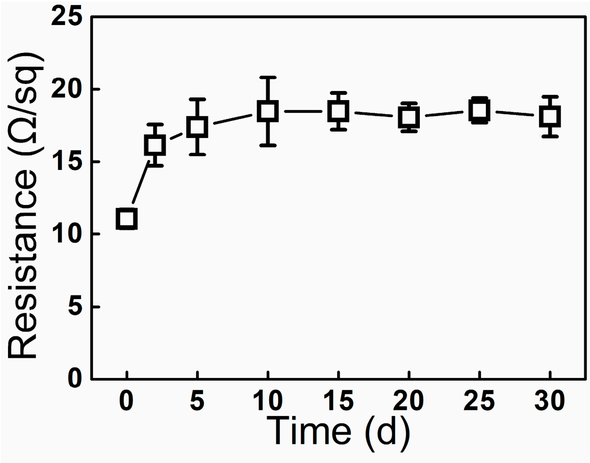

Figure S6. Sheet resistance of the Ga-PU composite with 25 vol% of Ga as a function of monitoring time under ambient condition.

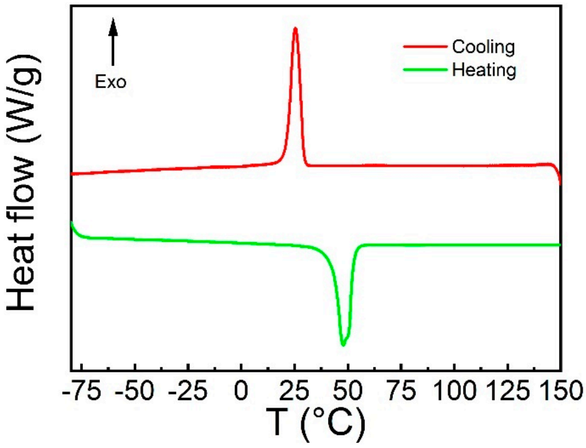

Figure S7. Differential scanning calorimetry (DSC) curve of the PCL-diol.

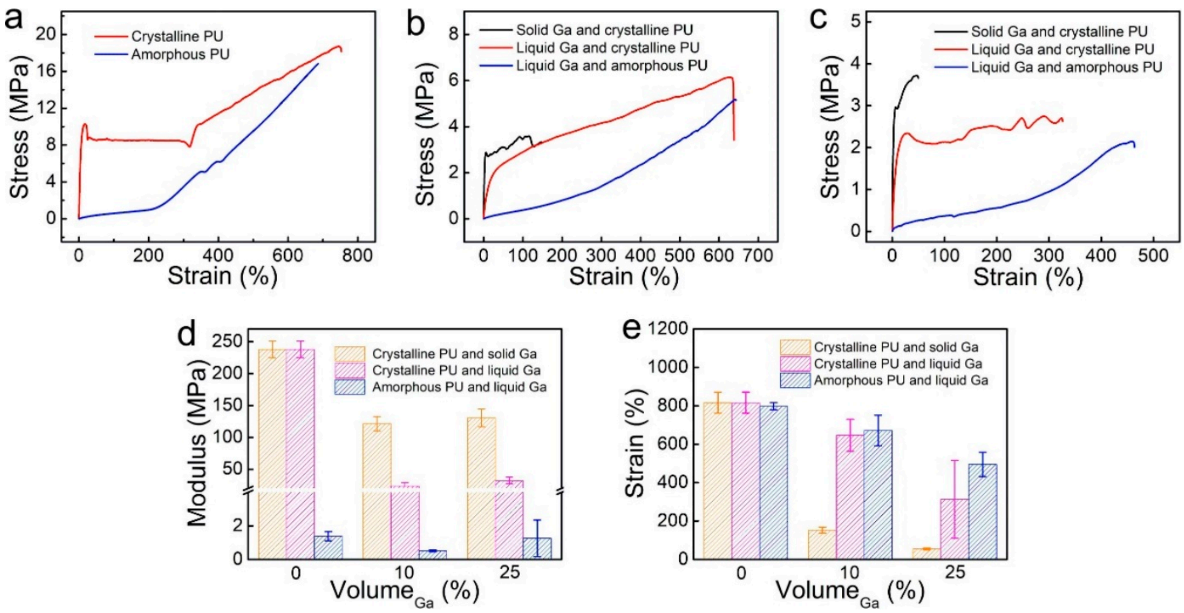

Figure S8. (a) Representative stress-strain curves of (a) a PU elastomer, (b) a Ga-PU composite with 10 vol% Ga, and (c) a Ga-PU composite with 25 vol% Ga. (d) Elastic modulus and (e) strain at break for a PU elastomer and a Ga-PU composites with 10 and 25 vol% Ga.

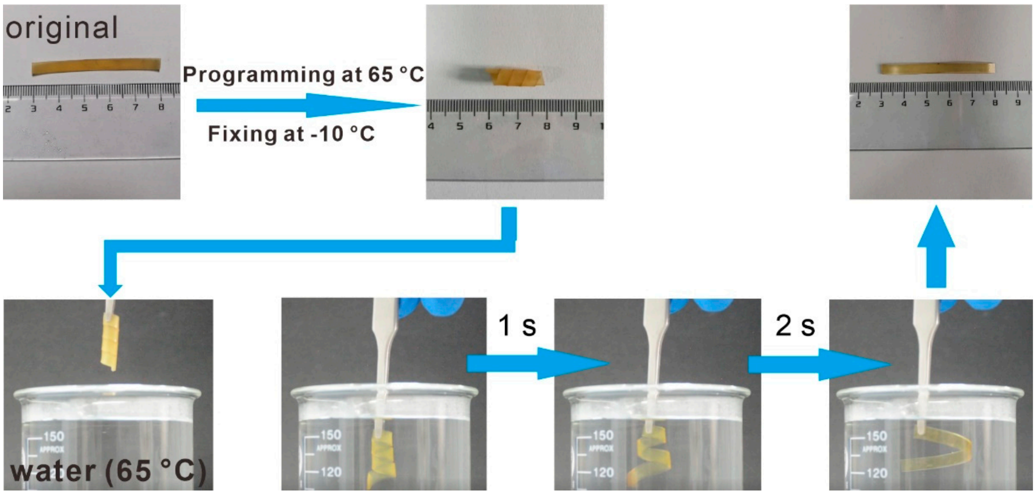

Figure S9. Shape programming and shape recovery of the pure PU elastomer. A strip of elastomer was programmed into curled shape at 65 °C, and then fixed at -10 °C. The shape recovery of the curled-shaped elastomer was conducted by immersing in 65 °C water.

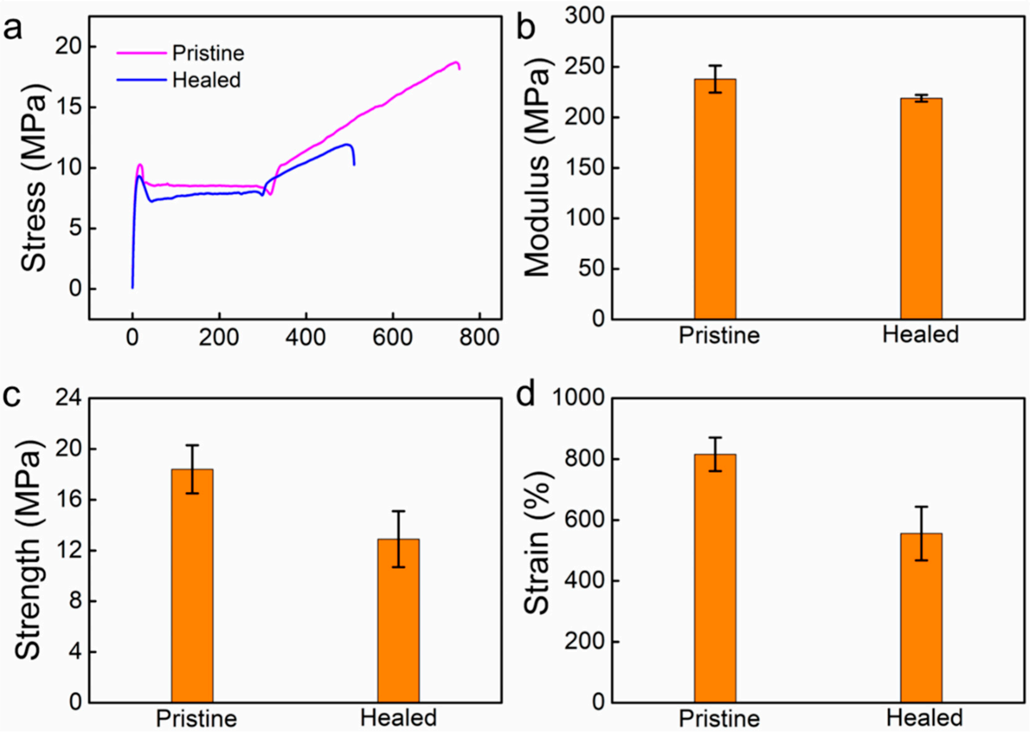

Figure S10. Mechanical properties of the pristine and healed PU elastomers. (a) The stress-strain curves, (b) the elastic modulus, (c) the strength at break, and (d) the strain at break for the pristine elastomer and the damaged elastomer healed at 120 °C for 10 minutes and 65 °C for 72 hours.
